# Increased severity of acute kidney injury does not increase long-term mortality

**DOI:** 10.1186/cc9522

**Published:** 2011-03-11

**Authors:** MB Pereira, DMT Zanetta, RCRM Abdulkader

**Affiliations:** 1 University of São Paulo, Brazil; 2 School of Medicine, São Paulo, Brazil

## Introduction

Acute kidney injury (AKI) increases either in-hospital mortality or long-term mortality. The severity of AKI has been associated with early mortality; however, studies on its role in long-term mortality have shown contradictory results.

## Methods

We studied 300 critically ill patients who had survived an AKI episode defined by AKIN creatinine criteria. All patients were attended by nephrologists in 2005 to 2006 and studied in May 2008. Exclusion criteria: age <18 years, presumed etiology other than acute tubular necrosis, baseline creatinine >3.5 mg/dl, nephrology follow-up <2 days and renal transplant. Analyzed variables: age; gender; type of admission; AKI etiology (ischemia, nephrotoxicity, sepsis or multifactorial); baseline and hospital discharge GFR (evaluated by MDRD equation); AKIN classification (1, 2, 3); need for dialysis, mechanical ventilation or vasoactive drugs; presence of comorbidities (hypertension, diabetes, heart failure, cancer or chronic liver disease); and functional recovery at hospital discharge (discharge GFR ≤1.1 baseline GFR). Survivors and nonsurvivors were compared by *t *test, Mann-Whitney test, Fischer's test or chi-square test, as appropriate. Causes of death were identified by death certificate. Data are presented as median (25 to 75 IQ) or percentage.

## Results

At the end of the study 105 patients had died (35%). Death occurred 194 days (69 to 444) after hospital discharge. The main cause of death was cardiovascular diseases (39%). The comparison between survivors and nonsurvivors showed that survivors had higher percentage of males (67 and 52%, *P *= 0.01), were younger (63 (49 to 72) and 70 years (56 to 79), *P *< 0.0001), had more multifactorial AKI etiology (26 and 41%, *P *= 0.01) and less heart failure as comorbidity (17 and 32%, *P *= 0.006). Unexpectedly, more survivors had needed mechanical ventilation (57 and 32%, *P *= 0.006) but neither vasoactive drugs (60 and 61%, *P *> 0.05) nor dialysis (38 and 39%, *P *> 0.05). See Table 1.

**Table 1:** AKI characteristics

	Survivors	Nonsurvivors	*P *value
AKIN 1; 2; 3 (%)	39; 25; 36	40; 30; 30	0.38
Baseline GFR	63 (41 to 88)	54 (33 to 74)	0.015
Hospital discharge GFR	53 (36 to 73)	42 (27 to 65)	0.02
Functional recovery (%)	49	47	0.8

## Conclusions

Long-term survival after AKI is not associated with the AKI severity but with baseline renal function.

